# Anti-Outer membrane protein C and anti-glycoprotein 2 antibodies in inflammatory bowel disease and their association with complicated forms of Crohn’s disease

**DOI:** 10.1186/s12876-014-0190-1

**Published:** 2014-12-31

**Authors:** Darina Kohoutova, Marcela Drahosova, Paula Moravkova, Stanislav Rejchrt, Jan Bures

**Affiliations:** 2nd Department of Internal Medicine - Gastroenterology, Charles University in Praha, Faculty of Medicine at Hradec Kralove, University Teaching Hospital, Sokolska 581, Hradec Kralove, 500 05 Czech Republic; Research Department of Cancer Biology, National Medical Laser Centre, University College London, 67-73 Riding House Street, London, W1W 7EJ UK; Department of Clinical Immunology and Allergology, Charles University in Praha, Faculty of Medicine at Hradec Kralove, University Teaching Hospital, Sokolska 581, Hradec Kralove, 500 05 Czech Republic

**Keywords:** Anti-OmpC antibodies, Anti-GP2 antibodies, Inflammatory bowel disease

## Abstract

**Background:**

Precise diagnostics of inflammatory bowel disease (IBD) and identification of potentially more aggressive phenotypes of Crohn’s disease (CD) is urgently needed. The aim of our prospective study was to assess the relationship between serum anti-OmpC IgA (Outer membrane protein C), anti-GP2 (anti-glycoprotein 2) IgG and anti-GP2 IgA antibodies with IBD and their association with complicated forms of CD.

**Methods:**

The study included 86 patients with CD, 25 patients with UC and 45 controls, blood donors. In CD group, 24/86 (28%) had B1 phenotype, 20/86 (23%) B2, 13/86 (15%) B3 and 29/86 (34%) B2 + B3. L1 involvement was present in 13/86 (15%), L2 in 13/86 (15%), L3 in 60/86 (70%). Serum anti-OmpC IgA, anti-GP2 IgG and IgA antibodies were investigated by means of ELISA. The data obtained were tested statistically by means of descriptive statistics, non-paired t-test, Mann-Whitney rank sum test, Spearman rank order correlation and Pearson product moment correlation using SigmaStat software.

**Results:**

Anti-OmpC IgA were noted to be significantly higher in CD (median 32.6, inter-quartile range (IQR) 18.9-60.7) compared to the controls (median 18.3, IQR 11.1-23.1), p < 0.001. Anti-GP2 IgG were significantly higher in CD (median 13.9, IQR 8.6-25.6) compared to the controls (median 8.0, IQR 4.7-10.8), p < 0.001. Anti-GP2 IgA were significantly higher in CD (median 20.1, IQR 9.1-40.4) compared to the controls (median 9.8, IQR 5.6-16.9), p < 0.001. Significant difference was found in anti-OmpC IgA between UC (median 26.2, IQR 20.2-36.4) and the controls (median 18.3, IQR 11.1-23.1), p < 0.001. In CD anti-OmpC IgA were significantly higher in B2 compared to B1: p = 0.041 and in B2 + B3 compared to B1: p = 0.036. Anti-GP2 IgA were significantly higher in B2 + B3 compared to B1: p = 0.009 and in B3 compared to B1: p = 0.029. In CD there was a significant difference in anti-OmpC IgA between patients with surgery and without surgery, p = 0.005.

**Conclusions:**

We have confirmed association between anti-OmpC IgA and IBD (CD and UC) and an association between anti-GP2 (IgG and IgA) and CD. Patients with complicated forms of CD have significantly higher levels of anti-OmpC IgA and anti-GP2 IgA.

## Background

Inflammatory bowel disease (IBD) with its increasing incidence represents an extraordinary problem in the developed countries. Precise diagnostics of IBD, differentiation of ulcerative colitis (UC) vs. Crohn’s disease (CD) and recognition of potentially more aggressive phenotypes of CD from the benign ones is urgently needed. The opportunity to identify subgroups of patients, who are more likely to develop complicated B2 (stricturing) and/or B3 (penetrating) and/or P (perianal) form of CD, which would be followed by an appropriate early therapeutic intervention, should prevent surgery, need of hospitalization and could have favourable personal and economical impacts as well [1-5]. Large intestinal microbiota play an important role in the etiopathogenesis of CD [6,7]. Recent studies have shown, that there is an association between disease onset, location, behaviour and serologic markers, from which some do reflect contribution of large intestinal microbiota to the development of CD [8-16]. A study performed by Papp et al. showed that the presence of auto-antibodies (including anti-OmpC) is associated with more complicated behaviour of the disease and the need for surgery in CD patients [11]. Similarly Mow et al. revealed that patients with anti-OmpC antibodies were increasingly likely to have internal perforating disease and at greater risk of requiring small bowel surgery [13]. Dubinsky et al. performed an important study within the pediatric population and discovered that if a child was positive in ≥1 of the antibodies tested (including anti-OmpC and ASCA), the probability of progression to internal penetrating and/or stricturing disease during the follow-up was higher, in comparison to those children who were not found to be positive for these antibodies [16]. Pancreatic antibodies have been isolated more recently and association mainly with CD patients has been shown [14].

The aim of our prospective study was to assess the relationship between anti-OmpC IgA (Outer membrane protein C), anti-GP2 (anti-glycoprotein 2) IgG and anti-GP2 IgA antibodies with IBD and their possible association with complicated forms of CD.

## Methods

### Patients

A total of 86 consecutive patients with CD (37 men, 49 women, aged 20–79, mean 43 ± 14), 25 patients with UC (9 men, 16 women, aged 20–74, mean 44 ± 16) and 45 controls, healthy blood donors (24 men, 21 women, aged 22–60, mean 41 ± 11) were enrolled into the prospective study. In the CD group 24/86 (28%) patients had nonstricturing-nonpenetrating (B1) form of CD, 20/86 (23%) stricturing (B2) phenotype, 13/86 (15%) penetrating (B3) phenotype and 29/86 (34%) stricturing + penetrating (B2 + B3) phenotype according to Montreal classification (ref.: 2 Silverberg). None of these patients had isolated perianal CD. Small bowel involvement (L1) was present in 13/86 (15%), colonic involvement (L2) in 13/86 (15%), ileocolonic involvement (L3) in 60/86 (70%), none of the CD patients had isolated involvement of the upper gastrointestinal tract (L4).

The duration of CD was 0–39 years, mean 15 ± 9; the duration of UC was 3–47 years, mean 14 ± 11. A total of 10 patients with CD were not on any therapy, 26 patients were on 5-aminosalicylates and 50 had immunosuppressive therapy (+/− corticosteroids, methotrexate, azathioprine, anti-TNF therapy). Within the UC patient cohort 1 patient was not on any treatment, 14 patients were receiving 5-aminosalicylates and 10 patients were treated with immunosuppresive therapy at the time of their blood tests.

The extraintestinal manifestations in patients with CD included musculoskeletal, dermatological, ocular, renal and hepatopancreatobiliary complications.

### Serum antibodies

Serum IgA anti-OmpC antibodies were investigated by means of ELISA (purchased from QUANTA Lite TM OMP Plus, INOVA Diagnostics, San Diego, USA). Values <20 U/mL were assessed as negative, values >25 U/mL were considered to be positive according to the manufacturer. Anti-GP2 IgG and IgA antibodies were investigated by means of ELISA (Generic Assays, Berlin, Germany), with negative results <15 U/mL and positive results >20 U/mL. The investigation of antibodies in the peripheral blood was performed between year 2010 and 2012; all patients included into the study have remained under the regular surveillance.

### Statistical analysis

Data obtained were tested statistically by means of descriptive statistics, non-paired t-test, Mann-Whitney rank sum test, Spearman rank order correlation and Pearson product moment correlation using SigmaStat software (Version 3.1, Jandel Corp., Erkrath, Germany).

### Ethical issues

All patients included in the study were notified with the necessary information and provided informed consent via a signed form. The project was approved by the Joint Ethical Committee (Charles University in Praha, Faculty of Medicine at Hradec Kralove & University Teaching Hospital Hradec Kralove). For all data obtained, all personal identification information was removed in compliance with the Czech laws for protection of confidentiality.

## Results

**Figure 1:**
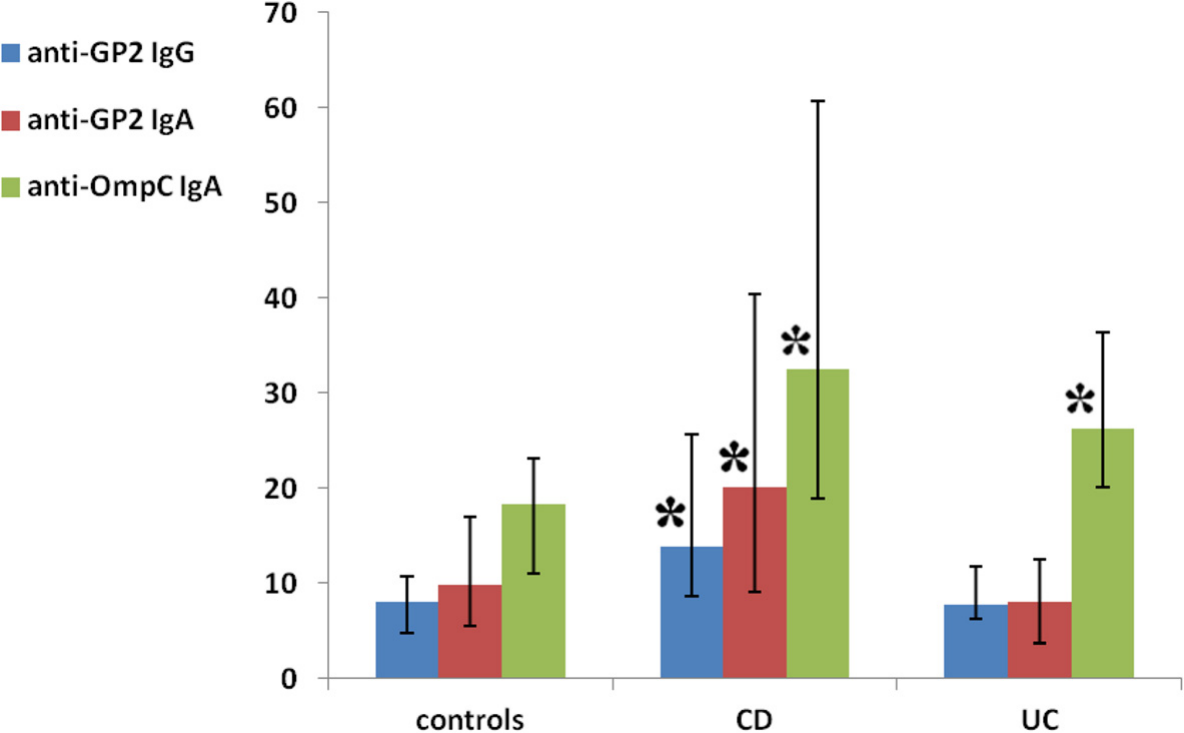
**Anti-GP2 IgG, anti-GP2 IgA and anti-OmpC IgA antibodies in controls, patients with CD and UC: median and inter-quartile range.** Asterisk (*) means statistically significant difference (p < 0.001) in relevant antibody between controls and CD/UC respectively.

Serum anti-OmpC IgA were significantly higher in CD (median 32.6, inter-quartile range (IQR) 18.9-60.7) compared to the controls (median 18.3, IQR 11.1-23.1), p < 0.001. Serum anti-GP2 IgG were also significantly higher in CD (median 13.9, IQR 8.6-25.6) compared to the controls (median 8.0, IQR 4.7-10.8), p < 0.001. Anti-GP2 IgA were significantly higher in CD (median 20.1, IQR 9.1-40.4) compared to the controls (median 9.8, IQR 5.6-16.9), p < 0.001, see Figure 1.

In CD there was a significant correlation between anti-GP2 IgG and anti-GP2 IgA values (r = 0.565, p < 0.001), between anti-GP2 IgG and anti-OmpC IgA values (r = 0.358, p < 0.001), between anti-GP2 IgA and anti-OmpC IgA values (r = 0.385, p < 0.001).

Table 1 provides percentige of abnormal values and values within normal ranges of serum anti-OmpC IgA, anti-GP2 IgG and anti-GP2 IgA in the controls, patients with CD and patients with UC.

**Table 1:** **Abnormal values and values within normal ranges of serum anti-OmpC IgA (>25U/mL vs. <20U/mL), anti-GP2 IgG (>20U/mL vs. <15U/mL) and anti-GP2 IgA (>20U/mL vs. <15U/mL) in controls, CD patients and UC patients**

	**controls**	**CD**	**UC**
**Anti-OmpC IgA >25U/mL**	8/45 (18%)	53/86 (62%)	13/25 (52%)
**Anti-OmpC IgA <20U/mL**	26/45 (58%)	22/86 (26%)	6/25 (24%)
**Anti-GP2 IgG >20U/mL**	1/45 (2%)	27/86 (31%)	4/25 (16%)
**Anti-GP2 IgG <15U/mL**	42/45 (93%)	49/86 (57%)	21/25 (84%)
**Anti-GP2 IgA >20U/mL**	8/45 (18%)	43/86 (50%)	2/25 (8%)
**Anti-GP2 IgA <15U/mL**	33/45 (73%)	35/86 (41%)	21/25 (84%)
**Sens/Spec/NPV/PPV of anti-OmpC IgA [%]**		71/76/54/87	68/76/81/62
**Sens/Spec/NPV/PPV of anti-GP2 IgG [%]**		36/98/47/96	16/98/67/80
**Sens/Spec/NPV/PPV of anti-GP2 IgA [%]**		55/80/49/84	9/80/61/20

Sens: sensitivity, spec: specificity, NPV: negative predictive value, PPV: positive predictive value.

Table 1 shows sensitivity, specificity, negative and positive predictive values of serum anti-OmpC IgA, anti-GP2 IgG and anti-GP2 IgA antibodies in each group of patients.

Descriptive statistics for each CD phenotype are given in Table 2. In CD Anti-OmpC IgA were significantly higher in B2 phenotype compared to B1: p = 0.041 and in B2 + B3 phenotype compared to B1: p = 0.036. Anti-GP2 IgA were significantly higher in B2 + B3 phenotype compared to B1: p = 0.009 and in B3 phenotype compared to B1: p = 0.029. Trend towards statistically higher levels of anti-GP2 IgG was observed in B2 + B3 when compared to B1 phenotype: p = 0.057.

**Table 2:** **Descriptive statistics (values of antibodies in U/mL) for each CD phenotype: B1: nonstricturing-nonpenetrating behaviour, B2: stricturing behaviour, B3: penetrating behaviour (Montreal classification)**

**Parameter**		**Median**	**Inter-quartile range**
**B1**	**anti-OmpC IgA**	21.0	16.0-42.5
	**anti-GP2 IgG**	11.9	7.0-15.5
	**anti-GP2 IgA**	11.1	6.3-24.5
**B2**	**anti-OmpC IgA**	40.9	25.1-54.8
	**anti-GP2 IgG**	18.0	8.3-48.1
	**anti-GP2 IgA**	16.6	6.4-37.3
**B3**	**anti-OmpC IgA**	33.2	17.7-74.6
	**anti-GP2 IgG**	11.9	8.7-20.4
	**anti-GP2 IgA**	30.5	13.3-48.2
**B2 + B3**	**anti-OmpC IgA**	38.7	23.5-67.3
	**anti-GP2 IgG**	16.1	10.5-33.3
	**anti-GP2 IgA**	21.6	14.8-65.7

Descriptive statistics for ileal (L1) CD, colonic (L2) CD and ileocolonic (L3) CD are provided in Table 3. Patients with L2 involvement had statistically significantly lower levels of anti-OmpC antibodies than patients with L3 involvement: p = 0.042. No other significant difference depending on location was found in anti-OmpC IgA, anti-GP2 IgG and anti-GP2 IgA.

**Table 3:** **Descriptive statistics (values of antibodies in U/mL): L1: involvement of terminal ileum, L2: colonic involvement, L3: ileocolonic involvement (Montreal classification)**

		**Median**	**Inter-quartile range**
**L1**	**anti-OmpC IgA**	27.8	17.7-49.2
	**anti-GP2 IgG**	11.5	9.6-33.3
	**anti-GP2 IgA**	22.3	7.4-39.6
**L2**	**anti-OmpC IgA**	18.8	15.4-32.7
	**anti-GP2 IgG**	10.6	7.7-14.9
	**anti-GP2 IgA**	22.2	6.6-39.0
**L3**	**anti-OmpC IgA**	38.2	22.4-63.9
	**anti-GP2 IgG**	14.8	7.6-29.2
	**anti-GP2 IgA**	20.1	10.5-42.8

Statistically significant difference was found in anti-OmpC IgA between UC (median 26.2, IQR 20.2-36.4) and the controls (median 18.3, IQR 11.1-23.1), p < 0.001, see Figure 1.

No statistically significant difference was found in serum anti-GP2 IgG levels between UC and the controls (p = 0.342) and in serum anti-GP2 IgA levels between UC and the controls (p = 0.138).

In UC there was a significant correlation between anti-GP2 IgA and anti-OmpC IgA values (r = 0.554, p = 0.004). No correlation was identified between anti-GP2 IgA and anti-GP2 IgG values, between anti-GP2 IgG and anti-OmpC IgA values.

No relationship was found between age and anti-OmpC IgA, anti-GP2 IgG, anti-GP2 IgA in CD patients and between age and anti-OmpC IgA in UC patients.

In CD there was no statistically significant difference in anti-OmpC IgA, anti-GP2 IgG, anti-GP2 IgA antibodies between groups of patients, who were diagnosed with CD ≤17 years (13/86, 15%), >17 and ≤40 years (64/86, 75%) and >40 years (9/86, 10%).

Weak but statistically significant negative correlation between the age of onset and anti-OmpC IgA was found in CD (r = −0.225; p = 0.037). No relationship was revealed between the duration of CD and value of anti-OmpC IgA, anti-GP2 IgG, anti-GP2 IgA antibodies respectively.

There were no differences in anti-OmpC IgA, anti-GP2 IgG, anti-GP2 IgA antibodies between patients with and without family history of IBD and with and without perinal disease in CD patients.

A trend towards statistically significant difference was revealed in anti-GP2 IgA between patients with an extraintestinal manifestation of CD (median 43.1, IQR 12.2-51.4) and without an extraintestinal manifestation of CD (median 17.5, IQR 8.8-34.4), p = 0.05. No statistically significant difference was found in anti-OmpC IgA or anti-GP2 IgG between patients with and without an extraintestinal manifestation of CD (p = 0.528; p = 0.588).

Significant difference in anti-GP2 IgA was found between CD patients who were either not on any therapy or were treated with 5-aminosalicylates only (median 11.9, IQR 7.3-33.9) and CD patients treated with +/− corticosteroids, methotrexate, azathioprine, anti-TNF therapy (median 25.6, IQR 11.7-46.8), p = 0.047. In CD there was no difference noted in anti-OmpC IgA, anti-GP2 IgG, anti-GP2 IgA antibodies between patients with anti-TNF and without anti-TNF therapy.

No relationship was revealed between disease onset, disease duration and anti-OmpC IgA, anti-GP2 IgG, anti-GP2 IgA antibodies in UC patients.

## Discussion

Mechanism of IBD development is still poorly understood, however it is generally accepted, that pathogenesis of IBD involves an inappropriate response of the mucosal immune system towards large intestinal microbiota in genetically susceptible individuals [17].

Until now, no exact bacterial species, which might be closely associated with the development of IBD, have been described. The main explanation could be, that only approximately 50% of intestinal bacteria are culturable [18]. Kotlowski et al. combined in their study culture-independent methods with bacterial culture and found out, that the tissue of IBD patients contained 3–4 logs higher amounts of bacteria from *Enterobacteriaceae* family and the difference between IBD patients and the controls was statistically significant. Also *Escherichia coli* genotypes B2 and D (which are associated with more virulent strains) were more prevalent in patients with IBD [19].

Anti-OmpC antibodies are aimed at porins, proteins embedded in the outer membrane of *Escherichia coli* [20]. Positivity of anti-OmpC antibodies in patients with CD has been described in a few recent studies [5,11,13]. Anti-OmpC antibodies were found in 55% of adult CD patients [21], 24% of pediatric CD patients, 11% of pediatric UC patients and 5% of the controls [22]. We found positivity of serum anti-OmpC IgA antibodies in 62% adult CD patients and in 52% adult UC patients, both results are significantly higher when compared to the controls. Eventhough immunoreactivity of anti-OmpC to pANCA antibodies was described [23], and therefore we could hypothesize anti-OmpC seropositivity in UC patients, our results do confirm closer relationship of anti-OmpC antibodies with CD than with UC. The above mentioned study carried out by Kotlowski et al. [19] with the combination of well-known specific association of adherent-invasive *Escherichia coli* with ileal mucosa in CD [24] explain our results.

Patients with complicated forms of CD - stricturing (B2) and stricturing + penetrating (B2 + B3) phenotype had significantly higher serum levels of anti-OmpC antibodies when compared to those with nonstricturing-nonpenetrating (B1) phenotype in our study and patients with ileocolonic involvement (L3) had higher levels of anti-OmpC compared to patients with isolated colonic (L2) involvement. Association of anti-OmpC antibodies with complicated forms of CD - internal penetrating disease (B3) and need for surgery has been published by other authors [10,13,14]. Dubinski et al. confirmed, that serum anti-OmpC antibodies are associated with internal penetrating and/or stricturing behaviour in the pediatric CD population [16].

Clear association of anti-OmpC antibodies with IBD, especially CD and complicated forms of CD highlights contribution of large intestinal microbiota to the etiopathogenesis of IBD. If the dysbiosis as a trigger of IBD pathogenesis could be influenced, we would be able to combat these diseases more successfully [25-27].

Anti-GP2 antibodies are aimed at GP2, which are specific receptors present not only in the exocrine pancreas, but also on microfold cells of intestinal Peyer’s patches, which are believed to be the hotbed of CD inflammation [28]. Association between anti-GP2 antibodies and CD has already been described [28-32] and we have confirmed the relationship between both, anti-GP2 IgG, anti-GP2 IgA with CD. Our patients with UC showed no difference in neither anti-GP2 IgG nor in anti-GP2 IgA from healthy controls, which is in agreement with data provided by Bogdanos et al. [32].

Higher prevalence of anti-GP2 antibodies in patients with complicated forms of CD has been postulated recently. Bogdanos et al. [32] revealed the association between anti-GP2 IgG with stricturing behaviour (B2) and perianal disease in CD patients. An extensive Hungarian study, which included 579 CD patients, confirmed the relationship of pancreatic antibodies with penetrating (B3) phenotype and perianal disease [31]. Our results are in close agreement with the Hungarian study: serum anti-GP2 IgA antibodies in our CD patients with B2 + B3 and B3 phenotype were characterised by significantly higher levels than in CD patients with B1 behaviour. We also found a trend towards statistically significant difference in anti-GP2 IgG between B1 and B2 + B3 phenotype.

Association between pancreatic antibodies and extraintestinal manifestation of CD was described by Lakatos et al. [31] and we also have confirmed higher levels of anti-GP2 IgA in CD patients, who have had an extraintestinal manifestation of the disease, however no statistically significant difference was noted.

According to the localisation of GP2 receptors in the small bowel Pavlidis et al. assumed anti-GP2 seropositivity in patients with ileal/ileocolonic CD, what he also confirmed [33]. On the contrary, we did not find any significant differences in anti-GP2 IgA and anti-GP2 IgG depending on disease location in our CD patients, however, the number of patients included might not be robust enough to draw any firm conclusions in this case.

The need of immunosupresive therapy in our patients (corticosteroids +/− methotrexate +/− azathioprine +/− biological therapy) was associted with higher levels of anti-GP2 IgA.

The serological markers, anti-OmpC and anti-GP2 antibodies, may aid us in diagnosing accurately the patients who have developed complicated forms of CD. By utilising these markers in clinical practice this could facilitate and guide us in forming tailored treatment plans for our patients.

## Conclusions

Association of anti-OmpC IgA with CD and UC was confirmed. Positivity of anti-OmpC IgA is more frequent in CD compared to UC.

CD patients with stricturing (B2) and stricturing + penetrating (B2 + B3) phenotype had significantly higher levels of anti-OmpC IgA compared to patients with nonstricturing-nonpenetrating (B1) phenotype.

Association between the need of surgery in CD patients and higher levels of anti-OmpC antibodies was revealed.

Relationship of anti-GP2 IgA and anti-GP2 IgG with CD but not with UC was found out.

CD patients with penetrating (B3) and stricturing + penetrating (B2 + B3) phenotype had significantly higher levels of anti-GP2 IgA compared to patients with B1 phenotype.

In CD higher levels of anti-GP2 IgA antibodies were found in patients with extraintestinal manifestation and in those who needed immunosuppressive therapy.
